# Validation of a Questionnaire for Distinguishing X-Linked Dystonia Parkinsonism From Its Mimics

**DOI:** 10.3389/fneur.2018.00830

**Published:** 2018-10-15

**Authors:** Jose Danilo B. Diestro, Mark Angelo C. Ang, Mark Willy L. Mondia, Paul Matthew D. Pasco

**Affiliations:** ^1^Department of Neurosciences, College of Medicine and Philippine General Hospital, University of the Philippines Manila, Manila, Philippines; ^2^Department of Laboratories, College of Medicine and Philippine General Hospital, University of the Philippines Manila, Manila, Philippines

**Keywords:** XDP, dystonia, questionnaire, parkinsonism, genetic, prevalence, epidemiology

## Abstract

**Objectives:** X-linked dystonia parkinsonism (XDP) is a neurodegenerative movement disorder endemic to the island of Panay in the Philippines. We undertook a population-based prevalence study to enumerate all cases of XDP in Panay. We first developed a 4-item questionnaire to distinguish XDP suspects from the general population. In the present study we aimed to revalidate this questionnaire to distinguish XDP from similar conditions so as to give it greater utility in the clinical setting.

**Patients and Methods:** A total of 306 subjects (114 cases and 192 controls) were screened in from the 16 towns and 1 city of Capiz province. Their responses to the previously developed 4-item questionnaire were collected and multivariable logistic regression was performed to develop a predictive model. The accuracy of the model was determined by using it on a subset of patients; then, a scoring system based on the model coefficients was established.

**Results:** With a cut-off score of 6, the questionnaire had an accuracy of 70.7% (95% CI 0.57-0.82), a sensitivity of 84.6 % (95% CI 0.65-0.96) and a specificity of 59.4 % (95% CI 0.41-0.76). The item on “shuffling of feet” was the strongest predictor in distinguishing XDP from its common mimics.

**Conclusion:** We were able to revalidate a simple, four-item questionnaire that could distinguish XDP from its common mimics with fair accuracy. The questionnaire along with other clinical features can be used to determine which patients need specialty evaluation and genetic testing to verify a diagnosis of XDP.

## Introduction

X-linked dystonia parkinsonism (XDP) is a neurodegenerative movement disorder endemic to Panay island in the Philippines, with recessive inheritance and a genetic founder effect. Molecular genetic studies suggest reduced expression of a neural isoform of *TAF1* in the striatum of patients (1). The disease initially manifests with focal dystonia, which then progresses to generalized dystonia and overlaps with symptoms of parkinsonism about 5–10 years after onset. Parkinsonism predominates after about 10 years of disease (2). Some XDP patients have may have predominantly parkinsonian symptoms initially (3), causing diagnostic confusion with idiopathic Parkinson Disease. Among inherited forms of isolated dystonia, XDP stands out with its overlapping parkinsonian features and X-linked mode of inheritance (4). The disease was brought to the attention of the international scientific community in 1975 when it was presented in the Second Dystonia Meeting held in New York City (5).

A population-based prevalence study was initiated in 2016 in order to do a complete enumeration of all XDP cases in Panay. The first phase involved the development of a 4-item screening questionnaire that can be used by community health workers (CHWs). The second phase involved screening by the CHWs of all the households in the island using this questionnaire. Individuals who were screened in were then evaluated by neurologists and movement disorder specialists. All consenting patients deemed to have probable XDP underwent genetic testing. Only Capiz province in Panay island has undergone all four phases of the study.

The first phase of the prevalence study was accomplished in 2015. A questionnaire with four items was validated to be highly sensitive and specific for screening patients with possible XDP in a general population where XDP is endemic. The questions screened for sustained twisting, repetitive jaw opening and closing, slowness in movement, and feet shuffling (6). Since the questionnaire was designed for screening at-risk individuals in a healthy population, the validity of this tool is unknown in patients with symptoms similar to XDP, such as Parkinson's disease, stroke, and cerebral palsy (6). A questionnaire that can distinguish genetically confirmed XDP from its clinical mimics is therefore lacking.

New diagnostic criteria for another genetic movement disorder, Huntington Disease, have recently been established. The criteria are based on motor scoring systems, genetic testing and historical findings that classify the patients into six strata (7). Similarly, a questionnaire for another neurologic condition, myotonic dystrophy (MD), identified 5 questions predictive of MD among healthy controls and diseases that mimic MD. The study emphasized the importance of such questionnaires especially in neurologic conditions such as MD that may be missed by non-neurologists (8). Common neurologic diseases, such as stroke, migraine, dementia and Parkinson disease also have screening tools used by non-specialists that lead to more definitive investigations (9–12).

The main objective of our study is to develop a weighted questionnaire that will differentiate XDP from other neurological diseases with similar symptoms. Many patients reside in remote areas of Panay, accessible only by long treks on foot and boat rides. General health professionals who are first to see patients with possible XDP will need to know which ones will need specialist assessment. The present questionnaire, if validated, can help direct meager community resources toward diagnosing and treating patients most likely to have XDP.

## Materials and methods

The study design involved the development of a predictive model using retrospectively obtained data from the ongoing Population-Based Prevalence Study of XDP in Panay. Over a period of 2 months, data was gathered from all the 16 towns and 1 city of Capiz province. The CHWs screened the entire province of about 440,000 adults^1^ using the previously validated 4-item questionnaire. In brief, the questionnaire was adapted from existing screening tools and tested on a random sample of 64 genetically confirmed and 64 normal healthy individuals (7). Responses to the questionnaire, the clinical diagnoses made by the movement disease specialists and the results of genetic testing provided data for the current study. Multivariable logistic regression was performed on a training subset of the data (*n* = 248) to create a predictive model and scoring system. The predictive model's performance was evaluated by applying the scoring system on another subset of data (*n* = 56). Sensitivity, specificity, and accuracy of the predictive model were obtained with 95% CI.

To be included in the dataset, subjects needed to be patients of the ongoing Population Based Prevalence Study of XDP in Panay, screened in by the CHWs in the second phase of the study. The subjects had a “yes” answer to at least one question in the 4-item questionnaire and a final disposition which is either a diagnosis of XDP or an alternative diagnosis, based on results of clinical and genetic testing.

Cases were defined as subjects with at least one positive response to the 4-item questionnaire and a positive genetic test result for XDP with the SVA retrotransposon insertion using a polymerase chain reaction (PCR) based genetic test (13). On the other hand, controls were defined as subjects with at least one positive response to the 4-item questionnaire and one of the following: a negative genetic test result or a more likely alternative diagnosis (in which case genetic testing was no longer done).

The patient's age, sex, and responses to the 4-item questionnaire, final clinical diagnosis and genetic testing results, where available, were tabulated on data collection sheets and subsequently, tabulated in an Excel file (Microsoft Excel for Mac v15.35). Anonymity was maintained by ensuring that only the codes were used in data gathering.

The collected data was randomly partitioned into two: a training set and a test set. The training set (248 subjects: 88 cases and 160 controls) was used to develop a predictive model through multivariable logistic regression analysis, which was appropriate to model the data because the output variable (whether or not a subject has XDP) was based on a set of predictor variables (responses to the screening questions) Each of the four questions in the screening tool was assigned a score based on the raw coefficients of the logistic regression model. A simulated dataset consisting of a list of all the possible combinations of answers to the four questions was used to determine the total score cut-off value. The resulting model was then run on the test set (58 subjects: 26 cases and 32 controls) to estimate its prediction abilities (diagnostic accuracy). The predicted probability cut-off value used in the estimation of the diagnostic accuracy, sensitivity, and specificity was chosen as the point on the ROC curve that maximizes sensitivity and specificity.

**Figure 1:**
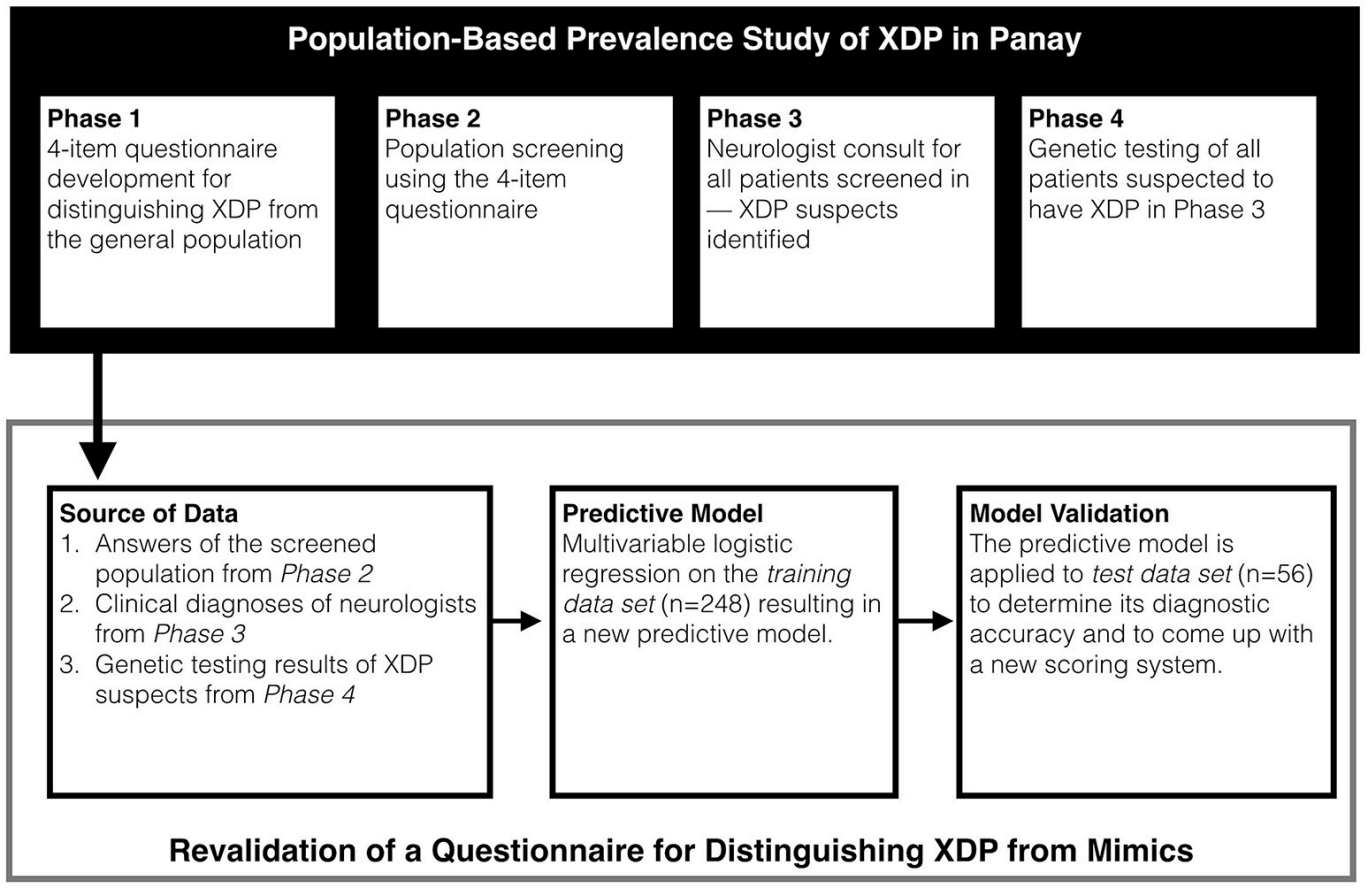
Study design. Data from the current study was taken from the ongoing Population Based Prevalence Study.

To allow for the predictive model to be used at the point of clinical encounter, a scoring system for the 4-item questionnaire was developed based on the model coefficients of the logistic regression model. All calculations and statistical analysis were done in R version 3.2.4 (www.R-project.org). Figure 1 provides a graphic summary of the experimental design.

The identity of all the subjects were concealed throughout the process. They were only referred to by their codes. The study obtained approval from the Expanded Health Research Office of the Philippine General Hospital prior to data collection. Also, a memorandum of agreement with the primary author of the Population Based Prevalence Study was signed to allow review of the study's de-identified data.

## Results

The baseline characteristics of all patients involved in the study is summarized in Table 1. Of all those seen by the CHWs during the second phase of the Population Based Prevalence Study, 306 subjects were screened in with an answer of “yes” to at least one item in the 4-item questionnaire. Out of all these patients, 114 had genetic confirmation of XDP, hence were labeled as cases. Of these, five were female. The average age was 53.14 years old (±10.79) with a range of 23 to 77 years old.

**Table 1:** Baseline Characteristics of Study Subjects.

**Characteristic**	**Value**
Total Number of patients	306
**MEAN AGE—YEARS (STANDARD DEVIATION)**	
All patients	47.6 (±18.3)
**SEX**	
No. of males (%)	229 (74.8)
No. of females (%)	77 (25.2)
**NUMBER OF PATIENTS WITH A “YES” RESPONSE TO:**	
Sustained twisting (%)	171 (55.9)
Repetitive jaw opening and closing	124 (40.5)
Slowness in movement (%)	209 (68.3)
Feet shuffling (%)	198 (64.7)
All four symptoms (%)	69 (22.5)

**Figure 2:**
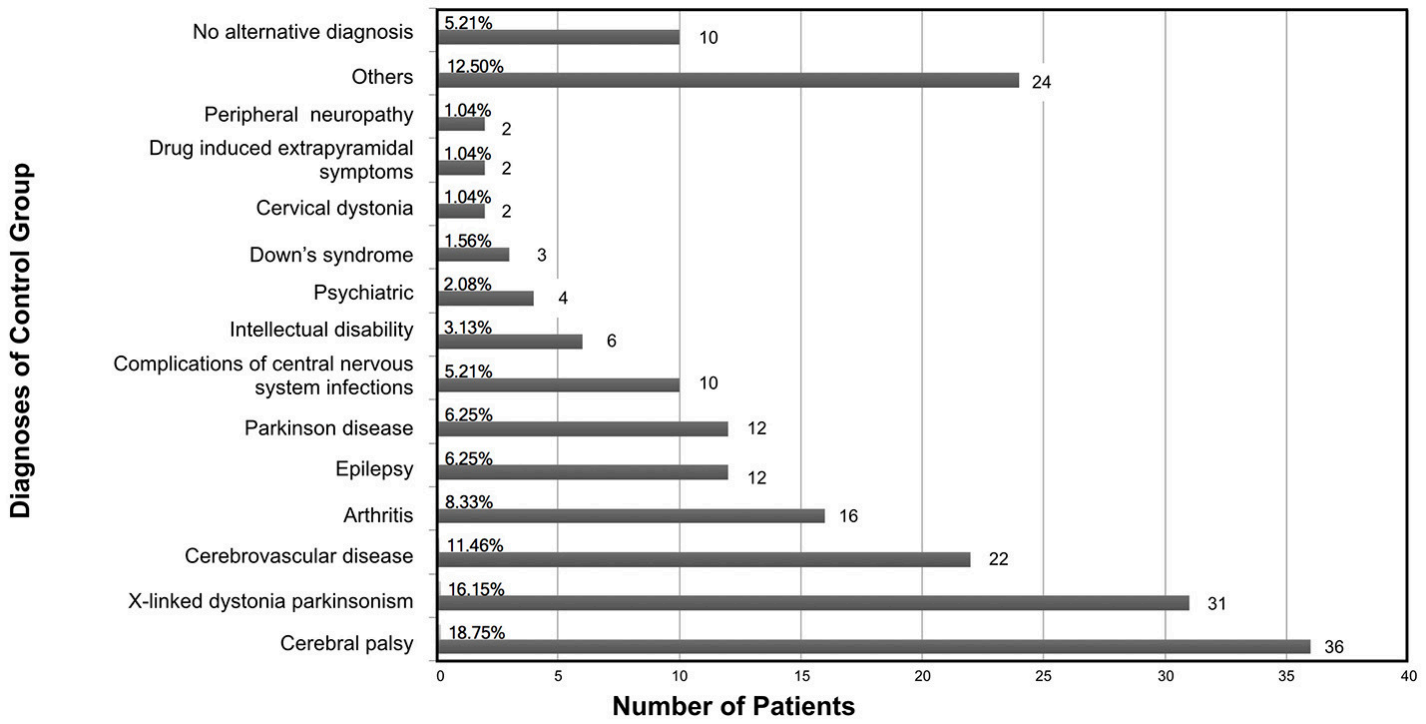
Breakdown of the diagnoses of the control group.

On the other hand, 192 either had a negative genetic test or a likelier alternative diagnosis after clinical evaluation, hence were labeled as controls. The diagnoses of the control group composed mainly of other neurologic diseases mimicking XDP are illustrated in Figure 2. Cerebral palsy (18.75%) was the most common XDP mimic. Forty one of the 192 patients in the control group also underwent genetic testing and had a negative result. Of the 12 Parkinson Disease patients, all in the control group, 5 underwent genetic testing and tested negative.

**Figure 3:**
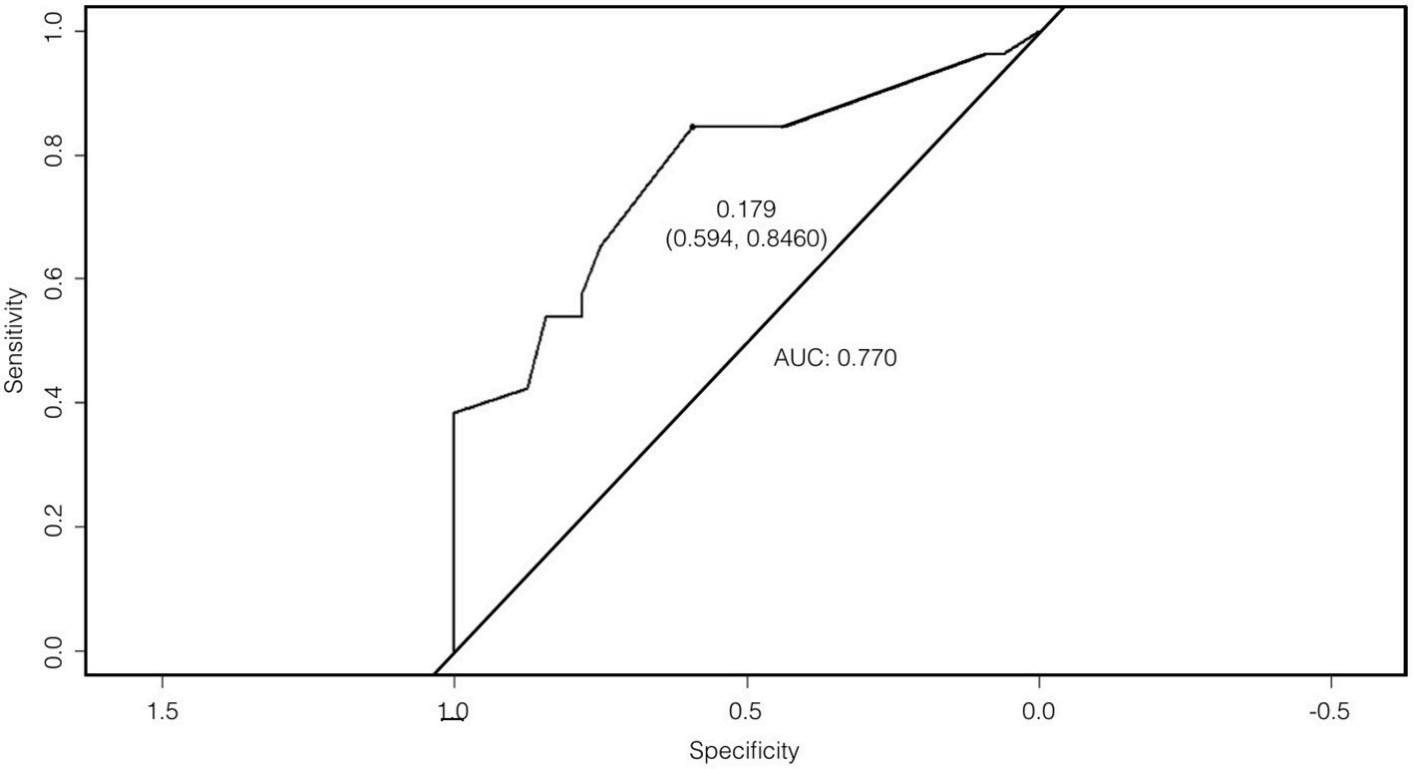
Receiver operating characteristic (ROC) curve with an area under the curve (AUC) of 0.770.

Based on the logistic regression model, the question on feet shuffling and taking smaller steps was found to be the single strongest predictor in distinguishing XDP from conditions that mimic it. The receiver operating characteristic (ROC) curve is shown in Figure 3. A cut-off probability of 0.179 for a “positive” result of the questionnaire was set, which was determined to be the best cut-off threshold maximizing the model's sensitivity and specificity. The area under the curve (AUC) is 0.770—reflecting a fair ability of the questionnaire to distinguish XDP from its mimics (14).

The final scoring system based on raw coefficients of the logistic regression model is shown in Table 2. The total scores for each combination of answers were compared with the predicted probabilities generated by running the model on the simulated dataset. The predicted probabilities of the simulated dataset are shown in Supplementary Material. The total score can range from 0 to 13. A score of 6 or more is considered “positive” i.e., the patient has a high likelihood of having XDP.

**Table 2:** Questionnaire items with corresponding weights.

**Questions**	**Score**	**Coefficient**	**95% CI**	***p*-value**
1. Do you experience sustained twisting movements of any body part?	3	4.26	2.17	<0.001^*^
2. Do you experience constant jaw opening and closing?	3	3.20	1.64	0.001^*^
3. Do you experience slowness in movement?	2	1.51	0.59	0.392
4. Do you shuffle your feet or take smaller steps when you walk?	5	7.61	2.99	<0.001^*^

* *Statistically significant (p < 0.05)*.

The over-all accuracy of the model in separating XDP from non XDP individuals in the test set is 71%, which is statistically different from the accuracy that will be achieved if all subjects are classified as XDP without regard to their answer to the questions (no information rate). The model can correctly identify 85% of subjects with XDP as having XDP (sensitivity) and can correctly identify 59% of subjects without XDP as not having XDP (specificity). On the other hand, a positive identification made by the model will be correct 63% of the time (positive predictive value) and a negative identification made by the model will be correct 83% of the time (negative predictive value). The negative predictive value (NPV) of the test, 83%, ensures that most of those excluded by the test are true negatives.

## Discussion

In this study we attempted to validate a four-item questionnaire originally for use by lay workers to screen a population endemic for XDP, in a group of individuals with signs and symptoms that may be misdiagnosed as XDP by non-specialists. We found that though highly sensitive and specific in the general population, it was fairly accurate in this subset of XDP mimics.

Experience from the Population-Based Prevalence Study reveals that many patients who were suspected to have XDP could not even be brought to the municipal health centers for screening because of time and financial constraints. This emphasizes the great resources that need to be mobilized to get expert opinion for XDP suspects. Once this study ends, evaluation by a movement disorder specialist and genetic testing will become even more elusive. The questionnaire we developed can be used to screen for patients most likely to have XDP. Identifying this subset of patients will enable a more focused use of resources for those most likely to benefit. The questionnaire can be used by both lay workers and general health professionals.

Up to a quarter to one half of patients with dystonia may be misdiagnosed or undiagnosed (15). Even among neurologists the correct diagnosis of movement disorders may be difficult to make. The rate of accurate diagnosis correlates with the physician's experience—with movement disorder specialists arriving at the right diagnosis more consistentl (16). Unfortunately, in the Philippines, for a population of 100,981,437 (17), there are only about 10 movement disorder specialists with formal training. This questionnaire can be used to assist general health professionals and CHWs in Panay in selecting patients who are most likely to benefit from subspecialty consult and genetic testing.

The developed questionnaire has a fair ability to distinguish XDP from its common mimics with an accuracy of 71% (0.57-0.82, 95% CI). It has a relatively high negative predictive value of 83% (0.61-0.95, 95% CI), which makes it useful for first line health workers in selecting patients that need to be triaged for further work-up and evaluation in tertiary institutions with neurologists and movement disorder specialists. A patient with a score of 6 or more would likely benefit from further evaluation by a movement disorder specialist and subsequent genetic testing. While a score of less than 6 would identify patients who probably have an alternate diagnosis. The earlier validation study that distinguished XDP patients from the healthy general population only required that any one of the 4 identified questions be positive for the screened individual to move to the next phase of the study (6).

Perhaps the main differential diagnosis for XDP is idiopathic parkinsonism. Multivariable logistic regression analysis reveals that the question on feet shuffling and taking smaller steps has the biggest weight for determining XDP in this population but is not by itself able to distinguish between genetically positive XDP and its mimics. A previous review on the phenomenology of XDP revealed that only 5.7% of XDP patients begin with parkinsonian symptoms, including shuffling of gait, and that only 18.6% are predominantly parkinsonism throughout the course of the illness (5). This study suggests that shuffling of feet and parkinsonism may be more important than previously thought. In contrast, the original validation study for the 4-item questionnaire gave constant jaw opening and closing the largest weight for distinguishing XDP from the general population (6).

A screening questionnaire for Parkinsonism found the following questions most predictive: stiffness and rigidity in legs, tremor and shaking, troublesome arm swing and feet stuck to floor (18). It is interesting to note that two of these items relate to lower extremity mobility as well. This fact emphasizes the need for the other 3 components not found in the PD questionnaire to come up with a scoring system that can distinguish XDP from PD. The original version of the 4-item questionnaire found the question on “shuffling of feet” to have excellent positive and negative clinical utility in differentiating XDP from the normal population (6). It is not surprising then that the same symptom is found to be most important in differentiating XDP from its mimics.

The most interesting aspect of the results perhaps is the 31 clinically diagnosed XDP patients who had a negative genetic test. In this study we used a PCR-based test that detected the presence of the SVA retrotransposon insertion in intron 32 of the TATA binding protein factor 1 (*TAF1*) in the XDP critical region in Xq13.1 (13). This retrotransposon insertion however, is not the only variant associated with XDP in the XDP haplotype on the X chromosome. Six other variants have been identified: single-nucleotide changes (DSC1, DSC2, DSC3, DSC10, DSC12) and one 48-bp deletion (19), although previous data has shown that all these genetic variants are in tight linkage disequilibrium (i.e., no variant has been described in patients in isolation from the rest).

Theoretically, each of the 31 patients with clinical features consistent with XDP but a negative genetic test could have any of the other 6 genetic variants apart from the SVA retrotransposon insertion; however, a previous study that investigated the genetic features in 166 clinically diagnosed XDP patients found that all 7 disease-associated genetic variants always occurred together with each other in all but 5 individuals. These 5 patients, clinically diagnosed to have XDP, had none of the 7 genetic variants, and were classified as phenocopies (19). The 31 clinically diagnosed XDP patients that were negative for the XDP haplotype on genetic testing may harbor a yet unidentified genetic variation that may be common to the 5 phenocopies in the previous study. Unfortunately, details on the individual phenomenology of the XDP in these 31 patients apart from their answers to the questionnaire were not documented. We recommend that further clinical evaluation be done for these subjects. Features such as age of onset of the disease and initial neurologic manifestations may determine if this subset of patients has a phenotype that is unique from genetically confirmed XDP patients.

Further modifications to the questionnaire can be made that can utilize other questions from an expanded list of screening questions (6). The test-retest reliability of the questionnaire can also be explored in future studies. New questions can be added from focused group discussions involving movement disorder specialists, health workers and patients. A questionnaire with better diagnostic performance (i.e., higher sensitivity and specificity) can thus be produced. Involvement of stakeholders will also improve the acceptability of the questionnaire later. All these can help produce a more accurate and acceptable questionnaire. In addition, a convenient electronic application can also be developed.

Similar to developments in Parkinson Disease research, the principles of objectification, multi-purpose design and simplification should be applied to future assessment tools for XDP (20). Further modification of the tool can include an objective assessment of the pathologic movements of XDP by clinical observation or the use of wearable movement analysis devices. Doing so would make the evaluation adhere to an objective multipurpose design that gathers data for XDP phenomenology research (20).

There are several limitations of this study. The prevalence study only screened those able to understand and respond to a “yes or no” questionnaire-type interview. The new scoring tool was developed using subjects who had already been screened-in using the 4-item XDP screening questionnaire developed in phase 1 of the Prevalence Study. Only the same 4 questions were used to develop the new scoring tool. The questionnaire by itself cannot be used to decide which patients merit genetic testing. A careful evaluation by a movement disorder specialist is needed to elicit clinical clues that cannot be provided by the questionnaire.

All data was only collected through review of the database of an ongoing study. Because not all controls were genetically tested, some patients, especially those thought to have PD, may be XDP patients with predominant parkinsonian symptoms. Besides answers to the four questions no other clues to the patients' clinical features were found; hence, correlations between signs and symptoms to the genetic variant tested for could not be done. Most importantly, XDP patients without any of the currently known 7 mutations may not have been selected by the questionnaire.

## Conclusions and recommendations

We were able to revalidate a simple, four-item questionnaire that can be used to distinguish XDP from its common mimics with fair accuracy. The questionnaire is intended for use by local health workers and general practitioners to determine which patients need specialty evaluation and genetic testing to ascertain or dispute the diagnosis of XDP. Doing so would direct time and finances to the patients most likely to have XDP and need specialty care.

The clinical features of all subjects classified as cases should be more completely characterized. Doing so would contribute greatly to data regarding the phenomenology of XDP. Further genetic profiling should be done on the 31 clinically diagnosed XDP patients with negative genetic testing, as they could be harboring a yet to be discovered genetic mutation associated with XDP. A follow-up study that includes more controls with genetic testing may perhaps improve the accuracy of this questionnaire.

## Data availability statement

The raw data supporting the conclusions of this manuscript will be made available by the authors, without undue reservation, to any qualified researcher.

## Author contributions

JD, MM, MA, and PP were all equally responsible for the conception and design, acquisition of data or analysis and interpretation of data. MA designed and carried out the statistical analysis of the data. JD and MM drafted the article while MA and PP took charge of critical revisions for important intellectual content. JD, MM, MA, and PP agreed to be accountable for the article and to ensure that all questions integrity of the article are investigated and resolved.

## Funding

Collaborative Center for X-linked Dystonia Parkinsonism (CCXDP) of the Massachusetts General Hospital is responsible for the funding of the Population-based prevalence study of X-Linked Dystonia Parkinsonism (XDP) in Panay Island, an ongoing study whose database our raw data is from.

### Conflict of interest statement

The authors declare that the research was conducted in the absence of any commercial or financial relationships that could be construed as a potential conflict of interest.

## Footnotes

^1^Philippine Statistics Authority. *Capiz Quickstat*.
