# Corrigendum: Validation of a Questionnaire for Distinguishing X-Linked Dystonia Parkinsonism From Its Mimics

**DOI:** 10.3389/fneur.2018.00991

**Published:** 2018-11-20

**Authors:** Jose Danilo B. Diestro, Mark Angelo C. Ang, Mark Willy L. Mondia, Paul Matthew D. Pasco

**Affiliations:** ^1^Department of Neurosciences, College of Medicine and Philippine General Hospital, University of the Philippines Manila, Manila, Philippines; ^2^Department of Laboratories, College of Medicine and Philippine General Hospital, University of the Philippines Manila, Manila, Philippines

**Keywords:** XDP, dystonia, questionnaire, parkinsonism, genetic, prevalence, epidemiology

In the original article, we neglected to include the funder, Collaborative Center for X-linked Dystonia Parkinsonism (CCXDP) of the Massachusetts General Hospital. CCXDP is responsible for the funding of the Population-based prevalence study of X-Linked Dystonia Parkinsonism (XDP) in Panay Island, an ongoing study whose database our raw data is from.

In addition, we neglected to acknowledge the neurologists and genetic counselors who examined the patients of the study. The corrected Acknowledgment appears below:

We wish to acknowledge Dr. Roland Dominic Jamora for his review of the study design during the conception of this research. We would also like to thank the neurologists and genetic counselors who were instrumental in the implementation of this study. But most of all we are grateful to the health workers of Panay who bear the daily burden of caring for our XDP brothers and sisters. This research did not receive any specific grant from funding agencies in the public, commercial, or not-for-profit sectors.

The authors apologize for this error and state that this does not change the scientific conclusions of the article in any way. The original article has been updated.

## Conflict of interest statement

The authors declare that the research was conducted in the absence of any commercial or financial relationships that could be construed as a potential conflict of interest.

