# Supplementary material for: Validation of a Questionnaire for Distinguishing X-Linked Dystonia Parkinsonism From Its Mimics
Source: Front Neurol. 2018 Oct 15;9:830. doi: 10.3389/fneur.2018.00830 (PMC6196251; doi:10.3389/fneur.2018.00830)
Supplement: Supplementary file 1 [file Data_Sheet_1.PDF]

Supplementary Material

Validation of a Questionnaire for Distinguishing X-linked Dystonia Parkinsonism from its mimics

Dr. Jose Danilo B. Diestro, Dr. Mark Angelo C. Ang, Dr. Mark Willy L. Mondia, Dr. Paul Matthew D. Pasco

Correspondence: Dr. Jose Danilo B. Diestro
E-mail Address: jbdiestro@up.edu.ph

Supplementary File 2

Table: Scenarios

Scenarios	Q1: Sustained Twisting	Q2: Jaw Opening and Closing	Q3: Slowness in movement	Q4: Feet Shuffling	Model Cut-Off (>=0.179)
1	0	0	0	0	0.023
2	0	0	0	1	0.149
3	0	0	1	0	0.034
4	0	0	1	1	**0.209**
5	0	1	0	0	0.069
6	0	1	0	1	**0.359**
7	0	1	1	0	0.100
8	0	1	1	1	**0.458**
9	1	0	0	0	0.089
10	1	0	0	1	**0.428**
11	1	0	1	0	0.129
12	1	0	1	1	**0.530**
13	1	1	0	0	**0.239**
14	1	1	0	1	**0.705**
15	1	1	1	0	**0.321**
16	1	1	1	1	**0.783**

Shown in bold text are the scenarios that determine a subject as having a high likelihood of having Parkinson Disease
